# MOF-Confined Sub-2 nm Stable CsPbX_3_ Perovskite Quantum Dots

**DOI:** 10.3390/nano9081147

**Published:** 2019-08-10

**Authors:** Zhenxing Li, Chengcheng Yu, Yangyang Wen, Zhiting Wei, Junmei Chu, Xiaofei Xing, Xin Zhang, Mingliang Hu, Miao He

**Affiliations:** State Key Laboratory of Heavy Oil Processing, College of New Energy and Material, Beijing Key Laboratory of Biogas Upgrading Utilization, China University of Petroleum (Beijing), Beijing 102249, China

**Keywords:** MOF, sub-2 nm, CsPbX_3_ quantum dots, photoluminescence, stable

## Abstract

The metal halide with a perovskite structure has attracted significant attention due to its defect-tolerant photophysics and optoelectronic features. In particular, the all-inorganic metal halide perovskite quantum dots have potential for development in future applications. Sub-2 nm CsPbX_3_ (X = Cl, Br, and I) perovskite quantum dots were successfully fabricated by a MOF-confined strategy with a facile and simple route. The highly uniform microporous structure of MOF effectively restricted the CsPbX_3_ quantum dots aggregation in a synthetic process and endowed the obtained sub-2 nm CsPbX_3_ quantum dots with well-dispersed and excellent stability in ambient air without a capping agent. The photoluminescence emission spectra and lifetimes were not decayed after 60 days. The CsPbX_3_ quantum dots maintained size distribution stability in the air without any treatment. Because of the quantum confinement effect of CsPbX_3_ quantum dots, the absorption and photoluminescence (PL) emission peak were blue shifted to shorter wavelengths compare with bulk materials. Furthermore, this synthetic strategy provides a novel method in fabricating ultra-small photoluminescence quantum dots.

## 1. Introduction

Metal halides with a perovskite crystal structure have gained significant interest in multidisciplinary research areas owing to their outstanding photovoltaic and optoelectronic properties [1,2,3,4,5]. In particular, lead-based trihalides have enabled a whole new class of highly-efficient, low-cost, and solution processable light-harvesting and light-emitting devices [6,7,8]. Such compounds exbibit a broad tunable photoluminescence ranging from the ultraviolet (UV) to the near-infrared (NIR) region in the electromagnetic spectrum, high photoluminescence quantum yield (PLQY), and a narrow full width at half-maximum (FWHM), whose properties inspire more and more researchers to exploit these materials to be applied in high-efficiency solar cells, light-emitting diodes (LED), low threshold lasers, high-sensitivity photodetectors, and so on [9,10]. In contrast to the hybrid organic–inorganic metal halide perovskite, all-inorganic metal halide perovskite shows a narrower emission spectrum and remarkably higher environmental stability against environment moisture, oxygen, and heat [11,12,13]. Thus, the all-inorganic perovskite framework without a volatile organic component is highly desired in photovoltaic and optoelectronic devices [13]. Yip et al. reported that the power conversion efficiency (PCE) of all-inorganic CsPbI_2_Br perovskite solar cells is up to 14.6%, and the PCE loss is only 20% after being heated at 85 °C for 300 h [12]. Based on its inorganic nature, the high PLQY (>80%) of CsPbBr_3_ quantum dots solution was maintained more than 30 days, while the MAPbBr_3_ quantum dots solutions exhibited dramatically decreased PLQY (<10%) in less than 5 days [14].

In the context of optoelectronic applications and photoluminescence emission, the well-dispersed perovskite quantum dots show high quantum yields, and tunable light emission wavelength. The demonstration of these novel perovskite quantum dots opens a new way to designing optoelectronic devices, such as solar cells, displays, lasers and photodetectors. The photoluminescence emission can be easily adjusted through size control of quantum dots and subsequently through quantum confinement. Luther et al. reported that the α-CsPbI_3_ quantum dots films were obtained, and the cubic phase of CsPbI_3_ quantum dots can be stable for months in ambient air [11]. Such quantum dots films showed long-range electronic transport, which were used to fabricate a solar cell, and the open-circuit voltage and the efficiency were 1.23% and 10.77%, respectively.

To dates, many efforts have been made to prepare the all-inorganic metal halide perovskite quantum dots of various composition and size so that their band gap can be adjusted to match the desired spectrum [15,16,17]. Colloidal quantum dots have been synthesized by using various approach, for instance, hot injection method [15], ligand-assisted reprecipitation [18], microemulsion methods [19], and crystallization at room temperature [20]. Solution-based synthetic approach can make well-defined cesium lead halide perovskite quantum dots via a capping ligand-assisted strategy, but the material stability issues (sensitivity to water and air) and polydisperse nature of solution-based synthetic approach generally leads to larger size (more than 10 nm) perovskite quantum dots. Therefore, the ligand-free method to control the size of the perovskite quantum dots play an extremely important role of application of perovskite quantum dots in electronics and optics. Herein, we have successfully prepared sub-2 nm all-inorganic cesium lead halide perovskite (CsPbX_3_, X = Cl, Br, and I) quantum dots in porous copper-benzene-1,4-dicarboxylate (Cu-BDC) frameworks by a metal organic framework (MOF)-confined route that employs the confinement effect of the uniform porous structures of Cu-BDC [21,22,23]. The CsPbX_3_ quantum dots are confined within the Cu-BDC frameworks. The highly uniform microporous structure of Cu-BDC can effectively restrict the quantum dots aggregation in synthetic process and endow the obtained sub-2 nm CsPbX_3_ quantum dots with well-disperse and excellent stability in ambient air. It is noteworthy that the size distribution of these CsPbX_3_ quantum dots confined in Cu-BDC is well remained in ambient air without any post-treatment.

## 2. Materials and Methods

**Synthesis of pure Cu-BDC:** 0.530 g of Cu(NO_3_)_2_•6H_2_O was dissolved in 45 mL of N,N-Dimethylformamide (DMF), after stirring for 2 h, 0.362 g of p-phthalic acid was added and continued stirring for another 2 h. Then the mixture was transferred to a 50 mL Teflon-lined autoclave and reacted at 110 °C for 36 h. After the reaction, the mixture was washed with DMF and ethanol three times, and dried at 40 °C for 8 h in −0.1 MPa to obtain the blue solid powder.

**Preparation of CsPbCl_3_@Cu-BDC:** In a typical synthesis of CsPbCl_3_@Cu-BDC, CsCl (5.1 mg) and PbCl_2_ (8.3 mg) were dissolved into Dimethyl sulfoxide (DMSO, 2 mL), and the mixture stirring at room temperature overnight. Subsequently added 10 mg of Cu-BDC into perovskite precursor and stirred mixing for 2 min. The mixed solution was dried under −0.1 MPa at 80 °C for 30 min and then heat-treated at 150 °C for 0.5 h.

**Preparation of CsPbBr_3_@Cu-BDC:** In a typical synthesis of CsPbBr_3_@Cu-BDC, CsBr (6.4 mg) and PbBr_2_ (11 mg) were dissolved into DMF (2 mL), and the mixture stirring at room temperature overnight. Subsequently added 10 mg of Cu-BDC into perovskite precursor and stirred mixing for 2 min. The mixed solution was dried under −0.1 MPa at 80 °C for 30 min and then heat-treated at 150 °C for 0.5 h.

**Preparation of CsPbI_3_@Cu-BDC:** In a typical synthesis of CsPbI_3_@Cu-BDC, CsI (7.8 mg) and PbI_2_ (14 mg) were dissolved into DMF (2 mL), and the mixture stirring at room temperature overnight. Subsequently added 10 mg of Cu-BDC into perovskite precursor and stirred mixing for 2 min. The mixed solution was dried under −0.1 MPa at 80 °C for 30 min and then heat-treated at 150 °C for 0.5 h.

**Preparation of bulk CsPbX_3_ (X = Cl, Br, and I):** In a typical synthesis of bulk CsPbCl_3_, CsCl (5.1 mg) and PbCl_2_ (8.3 mg) were dissolved into DMSO (2 mL), and the mixture stirring at room temperature overnight. Subsequently the mixed solution were dried into powder under 300 °C. Similarly, the bulk CsPbBr_3_ and bulk CsPbI_3_ are synthesized in same way.

**Materials characterizations:** Transmission electron microscopy (TEM) images were carried out on a JEM 2100 LaB6 at 200 kV (Tokyo, Japan). The high-resolution transmission electron microscope (HRTEM) and energy-dispersive X-ray analysis (EDS) were showed on Tecnai F20 (Hillsboro, OS, USA) with an accelerating voltage at 200 kV. The wide-angle X-ray diffraction (XRD, Bruker D8 Advance instrument, Karlsruhe, Germany) patterns were recorded on a Burker D8-advance X-ray power diffractometer operated at 40 kV and current of 40 mA with Cu-Kα radiation (λ = 1.5406 Å). X-ray photoelectron spectrometer (XPS, Kanagawa, Japan) was carried out on an ion-pumped chamber (evacuated to 2 × 10^−9^ Torr) of an Escalad5 spectrometer, using Mg KR radiation (BE) 1253.6 eV. The PL spectrum, the photoluminescence quantum yield and the PL emission lifetime used fluorescence spectrometer (FLS980) from Techcomp (Beijing, China) Ltd. The UV-visible absorption spectrums were obtained using Jasco V-570 spectrometer (Shanghai, China). The laser Raman spectra were recorded on a Jobin-Yvon LabRAM HR800 Raman spectrometer (Paterson, SNJ, USA). Nitrogen sorption isotherms and pore size adsorption curves were determined at 77 K with a Micromeritics ASAP 2460 analyzer (Atlanta, GA, USA). Before the measurement, the samples were degassed in a vacuum at 300 °C for 6 h. The Brunauer–Emmett–Teller (BET) method was utilized to calculate the specific surface areas. By using the Barrett–Joyner–Halenda (BJH) model, the pore volumes and pore size distributions were derived from the adsorption branches of isotherms. The FT-IR spectra were determined at room temperature on a Perkin Elmer Frontier spectrometer (equipped with a DTGS detector). The elemental ratio were determined by ICP-MS (ICAP Q, Thermo, Waltham, MA, USA).

## 3. Results

**Figure 1:**
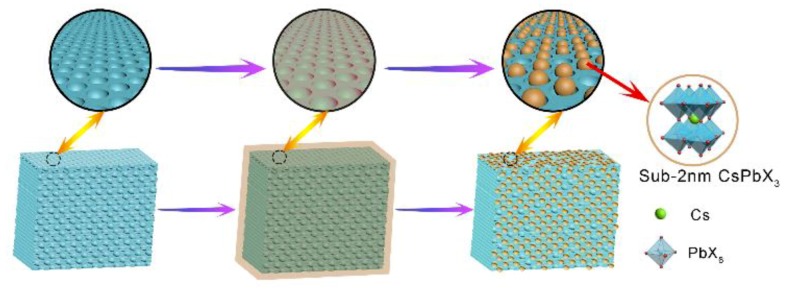
The schematic illustration about the synthetic process of metal organic framework (MOF)-confined CsPbX_3_ perovskite quantum dots.

The cesium halide and lead halide are served as the quantum dots precursors without adding any capping agent or ligand. The quantum dots precursor solution is absorbed into microporous structure of Cu-BDC by capillary force, so the obtained CsPbX_3_ perovskite quantum dots can be confined in Cu-BDC [24]. The direct preparation process is further schematically illustrated in Figure 1 to demonstrate the synthetic strategy of MOFs-confined CsPbX_3_ perovskite quantum dots.

The representative scanning electron microscopy (SEM) images of Cu-BDC can be seen in Figure S1a. The Fourier-transform infrared spectroscopy (FTIR) spectra (Figure S1b) showed in the broad band at 3000–3700 cm^−1^ indicates the presence of -OH groups and water, the peaks at 1576 cm^−1^ and at 1690 cm^−1^ correspond to the symmetric and asymmetric stretching vibrations of the carboxylate groups in Cu-BDC, respectively. Figure S2 shows X-ray diffraction (XRD) pattern of Cu-BDC, the diffraction peaks of the Cu-BDC fit very well with simulated Cu-BDC [25]. In Cu-BDC, terephthalate ligands are coordinated in a bidentate bridging fashion to a Cu^2+^. Each Cu^2+^ is also coordinated to a molecule of DMF to give the Cu^2+^ a square-pyramidal coordination geometry [25]. The Cu-BDC with uniform microporous was used to obtain sub-2 nm all-inorganic cesium lead halide perovskite quantum dots. The Brunauer–Emmett–Teller (BET) surface area of Cu-BDC was 512 m^2^/g (Figure S3a), and the pore-size distribution (Figure S3b) of Cu-BDC show the distinct peak at 0.68 nm, which indicates the presence of the microporous structure. Therefore, the Cu-BDC is ideal template to confine the perovskite quantum dots with an ultra-small size. In order to comparison our work, the pore size of Cu-BDC (Figure S4) can be obtained from the CIF standard of Cu-BDC (NO-687690) of the Cambridge Crystallographic Data Centre (CCDC), and the result is in line with the pore-size distribution curve.

**Figure 2:**
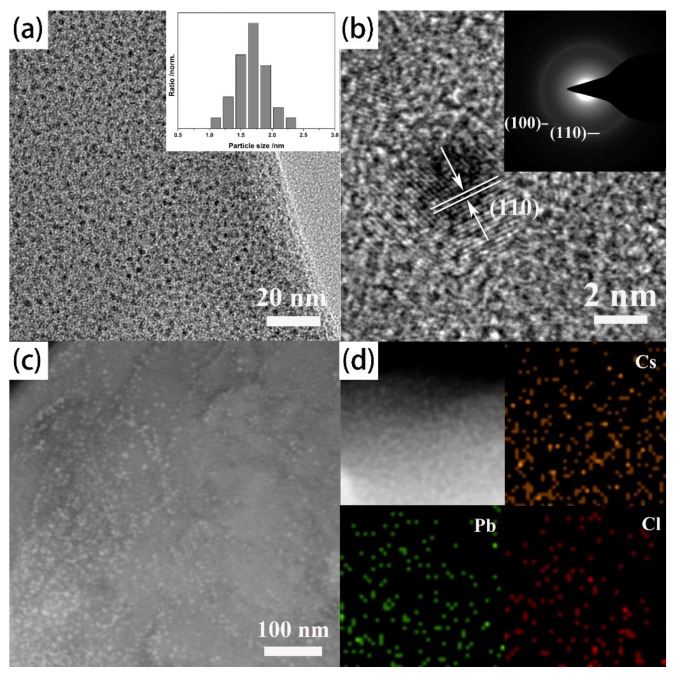
(**a**) The represent TEM image of CsPbCl_3_ perovskite quantum dots and narrow size distribution insets of figure. (**b**) The high-resolution transmission electron microscope (HRTEM) and selective area electron diffraction (SAED) images of CsPbCl_3_@Cu-BDC. (**c**) HAADF-STEM image of CsPbCl_3_ quantum dots. (**d**) The elemental mapping images showing the elemental distribution of Cs, Pb, and Cl.

Figure 2a shows the transmission electron microscopy (TEM) image of Cu-BDC confined CsPbCl_3_ perovskite quantum dots (CsPbCl_3_@Cu-BDC), with the average quantum dot size of 1.8 nm, and the extremely narrow size distribution is exhibited in the inset of the Figure 2a. The TEM examination reveals that the ultra-small 1.8 nm CsPbCl_3_ quantum dots are well embedded in the ordered pores of Cu-BDC. This effective confinement endows the as-obtained quantum dots with a uniform particle size. In addition, the high-resolution transmission electron microscope (HRTEM) displays a clear lattice spacing of 0.36 nm for the CsPbCl_3_ quantum dots in Figure 2b, which corresponds to the (110) facets of cubic perovskite phase. Further, the uniform dispersion of CsPbCl_3_ quantum dots within the Cu-BDC frameworks is further confirmed by the corresponding high-angle annular dark-field scanning transmission electron microscopy (HAADF-STEM) image as shown in Figure 2c. The bright spots, which are well dispersed in the Cu-BDC frameworks, are CsPbCl_3_ quantum dots. Additionally, the selective area electron diffraction (SAED) pattern of CsPbCl_3_@Cu-BDC further confirms that the CsPbCl_3_ quantum dots is the standard cubic perovskite phase (the inset in Figure 2b). The SAED rings represent the (100) and (110) facets of the cubic structure of CsPbCl_3_ pattern. The elemental mappings of the CsPbCl_3_@Cu-BDC are measured by energy-dispersive spectrometry (EDS) for Cs, Pb and Cl. Clearly, Cs, Pb and Cl are uniformly distributed throughout the Cu-BDC frameworks (Figure 2d). EDS point scanning experiments at arbitrary points reveal that Cs, Pb, and Cl are present with an atomic ratio of 1:1:3 (Figure S5 and Table S1), which further confirm that the ultra-small quantum dots are CsPbCl_3_ quantum dots.

**Figure 3:**
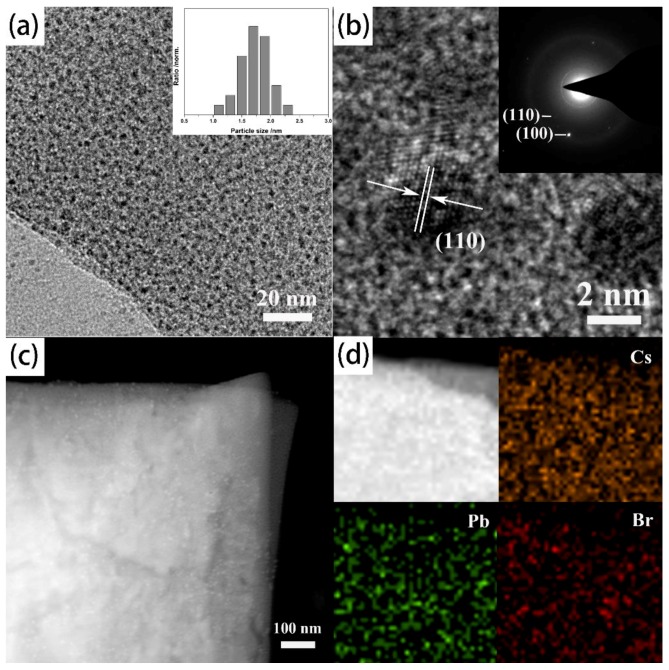
(**a**) The represent TEM image of CsPbBr_3_ perovskite quantum dots and narrow size distribution insets of figure. (**b**) The HRTEM and SAED images of CsPbBr_3_@Cu-BDC. (**c**) HAADF-STEM image of CsPbBr_3_ quantum dots. (**d**) The elemental mapping images showing the elemental distribution of Cs, Pb, and Br.

With a same method, the Cu-BDC confined CsPbBr_3_ perovskite quantum dots (CsPbBr_3_@Cu-BDC) with a very narrow size distribution is successfully prepared like process in Figure 3. For the average size of CsPbBr_3_@Cu-BDC quantum dot is 1.8 nm and the figure inset the Figure 3a is the narrow size distribution. The HRTEM about CsPbBr_3_@Cu-BDC is in Figure 3b which displays a clear lattice spacing of 0.41 nm for the CsPbBr_3_ quantum dots, and corresponds to the (110) facets of cubic perovskite phase. The SAED pattern inset Figure 3b is still of CsPbBr_3_@Cu-BDC, which confirmed the CsPbBr_3_ quantum dots is indicated the standard cubic perovskite phase. It also corresponds to the (100) and (110) planes of the cubic structure of CsPbBr_3_ pattern. The EDS is used to assess the elemental mappings of the CsPbBr_3_@Cu-BDC for Cs, Pb and Br. It can be observed that the Cs, Pb, and Br are distributed throughout the Cu-BDC frameworks uniformly (Figure 3c,d). And the point scanning by EDS experiments in random points illustrated that Cs, Pb and Br are present with an atomic ratio which is shown in Figure S6 and Table S1 is 1:1:3.

**Figure 4:**
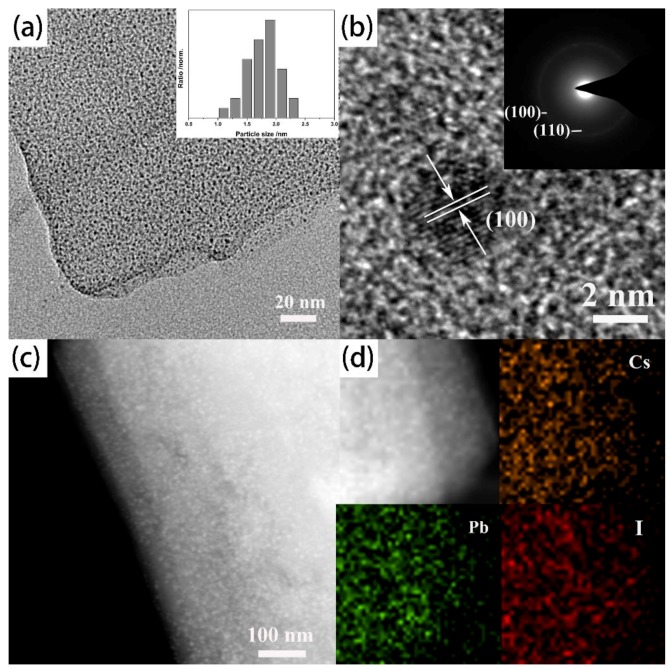
(**a**) The represent TEM image of CsPbI_3_ perovskite quantum dots and narrow size distribution insets of figure. (**b**) The HRTEM and SAED images of CsPbI_3_@Cu-BDC (**c**) HAADF-STEM image of CsPbI_3_ quantum dots, (**d**) The elemental mapping images showing the elemental distribution of Cs, Pb, and I.

The Cu-BDC confined CsPbI_3_ perovskite quantum dots (CsPbI_3_@Cu-BDC) is also achieved as shown in Figure 4. The average quantum dots size is 1.9 nm, and the narrow size distribution is showed in the inset of Figure 4a. The (110) facets of cubic perovskite phase can be inferred by the lattice spacing of 0.61 nm in the HRTEM Figure 4b. For further confirming the phase of CsPbI_3_@Cu-BDC, the SAED pattern (inset in Figure 4b) showed the CsPbI_3_ quantum dots is also the standard cubic perovskite phase like other CsPbX_3_. The SAED rings corresponds to the (100) and (110) facets, demonstrated the planes of the cubic structure of CsPbI_3_ pattern. The HAADF-STEM image and the corresponding elemental mapping images showed the elemental distributions of the CsPbI_3_@Cu-BDC about Cs, Pb, and I, which are well-distributed in the Cu-BDC frameworks (Figure 4c,d). As the other two samples, the atomic ratio also tested by EDS point scanning, the atomic ratio of Cs, Pb, and I are 1:1:3. (Figure S7 and Table S1).

In order to further confirm the above experimental results, X-ray photoelectron spectroscopy (XPS) characterization is used. The elemental ratio of CsPbCl_3_@Cu-BDC, CsPbBr_3_@Cu-BDC, CsPbI_3_@Cu-BDC for Cs:Pb:Cl, Cs:Pb:Br, and Cs:Pb:I measured by XPS amounts about 1:1:3, 1:1:3, and 1:1:3 (Figure S8 and Table S2), which match well the original molar ratio of the feed. The elemental ratio of XPS is in line with the result from inductively coupled plasma-atomic emission spectroscopy (ICP-AES, Table S3). From calculation of the ICP results, the percentage of the CsPbCl_3_, CsPbBr_3_ and CsPbI_3_ quantum dots loading in MOF pores is 17.56%, 18.39%, and 19.25%, respectively. Figure S9a shows that two strong peaks of CsPbCl_3_@Cu-BDC are located at about 138.9 eV (4f_7/2_) and about 143.8 eV (4f_5/2_) with a spin-orbit splitting energy of 4.9 eV which characteristic of Pb^2+^ states, and no metallic state of Pb^0^ is observed [26], and the 3d spectra of Cs showed there is only one type of Cs, and two signature peaks at 724.2 eV and 738.2 eV are Cs 3d_5/2_ and 3d_3/2_, respectively [27,28]. The core levels of Cl 2p in Figure S9a indicated the binding energy peaks at 199.1 eV and 197.8 eV are consistent with Cl 2p_1/2_ and 2p_3/2_ [29]. For CsPbBr_3_@Cu-BDC which shown in Figure S9b, can see the core levels of Br 3d binding energy are 77.3 eV for 3d_3/2_ and 74.9 eV for 3d_5/2_ suggests the Br^−^ state [30], meantime, the 143.3 eV for Pb 4f_5/2_, 138.4 eV for Pb 4f_7/2_, the 740.6 eV for Cs 3d_3/2_ and 726.6 eV for Cs 3d_5/2_. The Figure S9c is XPS for CsPbI_3_@Cu-BDC, and the binding energy of I 3d is 629.7 eV for 3d_3/2_ and 628.4 eV for 3d_5/2_ demonstrated there is only one type of I^−^ state [27]. Just like the other two samples, there are two peaks 737.5 eV, 723.6 eV for Cs 3d_3/2_ and Cs 3d_5/2_, two peaks 142.2 eV, 137.4 eV for Pb 4f_5/2_ and Pb 4f_7/2_ [26].

**Figure 5:**
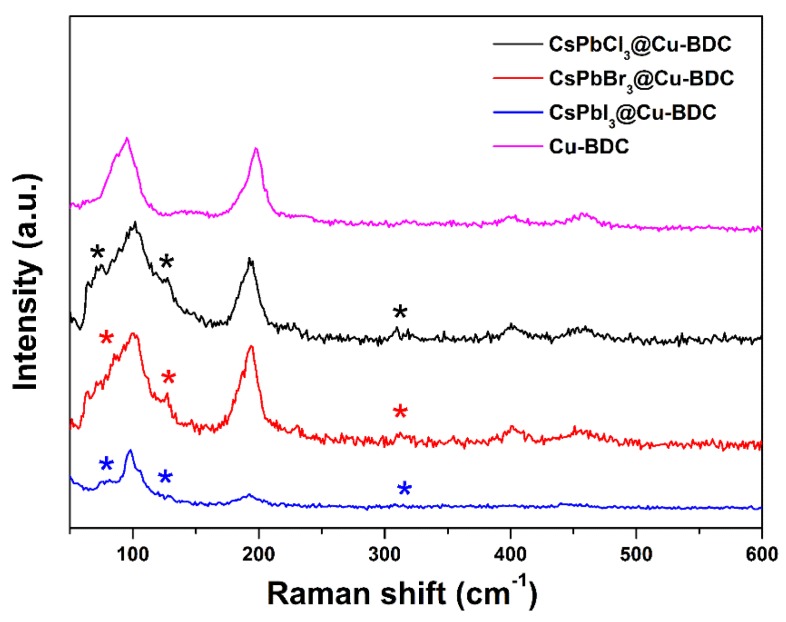
The Raman spectra of CsPbCl_3_@Cu-BDC, CsbBr_3_@Cu-BDC, CsPbI_3_@Cu-BDC and Cu-BDC. “*” represents the peaks.

The crystal structures of these three samples can be confirmed by Raman spectra. Figure 5 of Raman spectra excited by 633 nm laser light show a peak at 127 cm^−1^ and another peak at 82 cm^−1^, which is attributed to the vibrational mode of PbX_6_ octahedron and the motion of Cs^+^ [31,32]. A weak and broad band at 310 cm^−1^ is related to the second-order phonon mode of the octahedron. The crystal structure of CsPbX_3_ is confirmed to be perovskite structure [33]. The two strong peaks at 94 cm^−1^ and 197 cm^−1^ are attributed to Cu-BDC.

**Figure 6:**
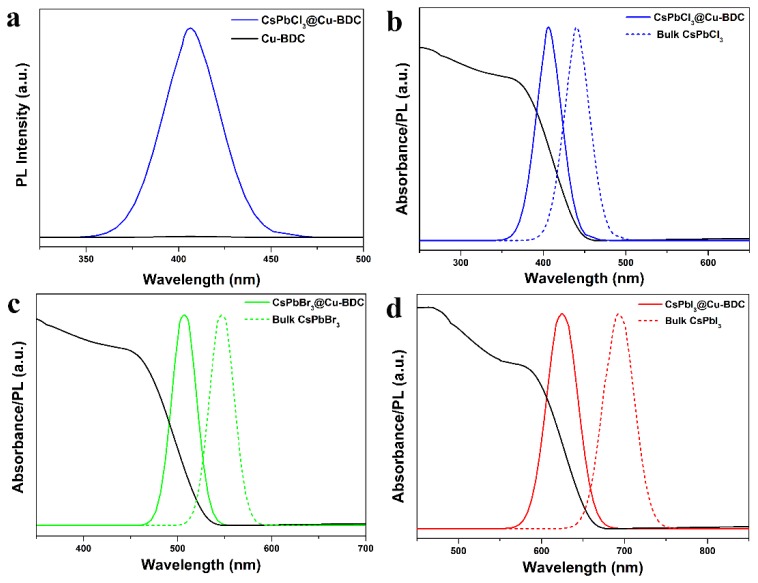
(**a**) PL spectrum of Cu-BDC and CsPbCl_3_@Cu-BDC; the UV-vis absorption spectrum and PL spectrum of (**b**) CsPbCl_3_@Cu-BDC and bulk CsPbCl_3_, (**c**) CsPbBr_3_@Cu-BDC and bulk CsPbBr_3_, and (**d**) CsPbI_3_@Cu-BDC and bulk CsPbI_3_.

The photophysical properties of Cu-BDC confined CsPbX_3_ perovskite quantum dots is investigated by ultraviolet-visible (UV-vis) absorption spectrum and PL spectrum measurements. The UV-vis absorption spectrum of CsPbCl_3_@Cu-BDC, CsPbBr_3_@Cu-BDC and CsPbI_3_@Cu-BDC is shown in Figure 6. Figure 6a shows the PL spectra of as-synthesized CsPbCl_3_@Cu-BDC and Cu-BDC powder. Obviously, Cu-BDC does not show any florescence signal in the visible range. The absorption peak is at 367 nm, 453 nm, and 579 nm, respectively, which is blue shifted shorter wavelengths from that of the bulk CsPbX_3_ (X = Cl, Br, and I), due to the quantum confinement effect of CsPbX_3_ (X = Cl, Br, and I). Figure 6b shows the PL emission spectrum of the CsPbCl_3_@Cu-BDC. The PL emission peak is at 406 nm, which is also blue shifted with ~32 nm compared with the bulk CsPbCl_3_ (438 nm). The full width at half-maximum (fwhm) is 38 nm. With regard to CsPbBr_3_@Cu-BDC, the obvious absorption peak at 453 nm is observed (Figure 6c), and the green PL emission peak is at 507 nm with fwhm of 32 nm, which is also blue shifted with ~39 nm compared with the bulk CsPbBr_3_ (546 nm). As shown in Figure 6d, the PL emission spectrum of the CsPbI_3_@Cu-BDC displays a red emission (624 nm), with a fwhm of 40 nm, which is also blue shifted with ~69 nm compared with the bulk CsPbI_3_ (693 nm). The absorption peak of the CsPbI_3_@Cu-BDC is at 580 nm. Compared with the previous work (J. Am. Chem. Soc. 2016, 138, 13,874−13,881), the distance between perovskite nanocrystals is extremely close (2 nm). Therefore, a smaller blue shift was attributed to the coupling between the perovskite nanocrystals [21].

**Figure 7:**
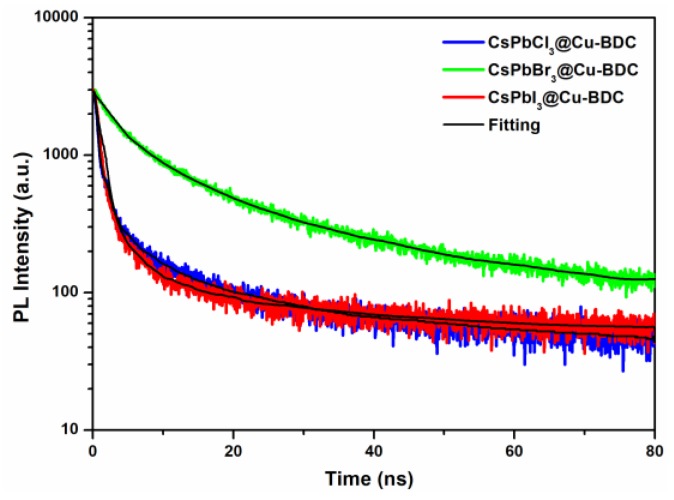
The photoluminescence (PL) emission decay curves of CsPbCl_3_@Cu-BDC, CsPbBr_3_@Cu-BDC, CsPbI_3_@Cu-BDC.

The PL emission lifetimes of these three perovskite quantum dots are studied by monitoring at the PL maximum emission wavelength of CsPbCl_3_@Cu-BDC, CsPbBr_3_@Cu-BDC and CsPbI_3_@Cu-BDC, showing the PL emission decay curves in Figure 7. The decay curves are analyzed to be best-fitted using the tri-exponential decay kinetics, and the kinetic parameters are summarized in Table S3. The short lifetime is concerned about the trap-assisted recombination at the boundary of quantum dots [34], while the long lifetime is related to the radiation recombination inside the quantum dots. The average PL lifetimes of CsPbCl_3_@Cu-BDC, CsPbBr_3_@Cu-BDC, and CsPbI_3_@Cu-BDC are 15.1, 24.4, and 18.75 ns, respectively. The absolute PLQY is measured by using commercial Hamamatsu setup. The absolute PLQY = N_emit_/N_absorb_, where N_emit_ is the number of emitted photon, and N_absorb_ is the number of absorbed photon. The absolute PLQY of CsPbCl_3_@Cu-BDC, CsPbBr_3_@Cu-BDC, and CsPbI_3_@Cu-BDC are 4.12%, 9.96%, and 18.3%, respectively (Table S4).

**Figure 8:**
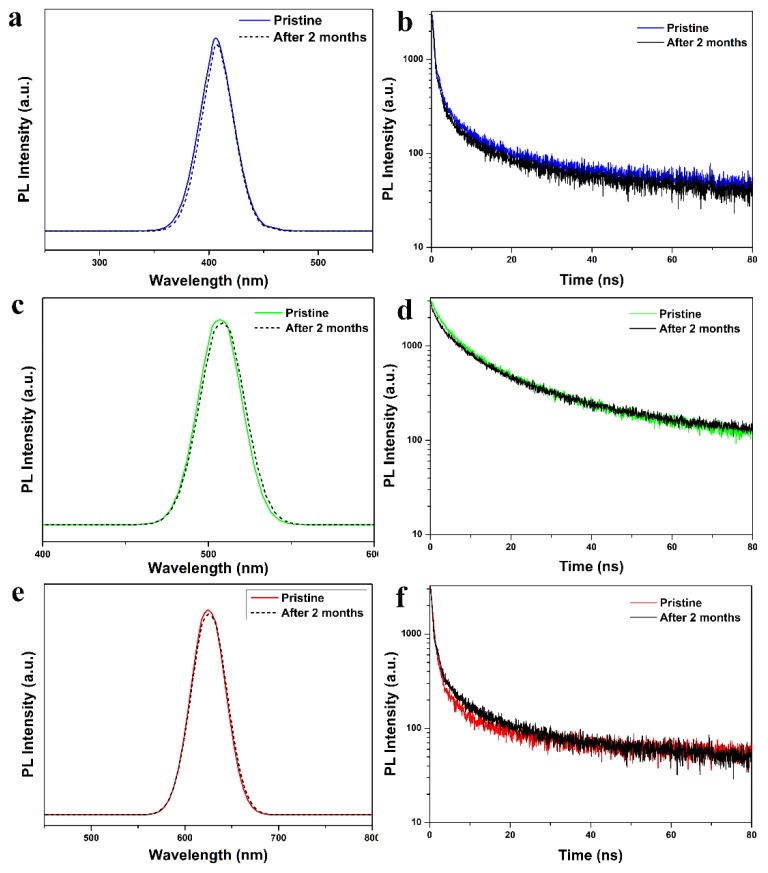
PL spectrum of pristine and after 60 days about (**a**) CsPbCl_3_@Cu-BDC, (**c**) CsPbBr_3_@Cu-BDC, and (**e**) CsPbI_3_@Cu-BDC. The PL emission decay curves of pristine and after 2 months about (**b**) CsPbCl_3_@Cu-BDC, (**d**) CsPbBr_3_@Cu-BDC, and (**f**) CsPbI_3_@Cu-BDC.

The PL emission of the CsPbCl_3_@Cu-BDC, CsPbBr_3_@Cu-BDC, and CsPbI_3_@Cu-BDC are very stable to environmental conditions because the perovskite (CsPbX_3_, X = Cl, Br, and I) quantum dots are embedded in Cu-BDC [35,36]. In order to test stability, the samples were kept under atmospheric conditions in the dark for 60 days. In these three samples, the PL emission spectra and lifetimes were not decayed after 60 days (Figure 8), and the PLQY of CsPbCl_3_@Cu-BDC, CsPbBr_3_@Cu-BDC, and CsPbI_3_@Cu-BDC is 4.08%, 9.72%, and 17.45%, respectively, which indicates the high stability at room temperature in air. It is well known that the CsPbX_3_ (X = Cl, Br, and I) bulk material spontaneously transitions from the perovskite phase to the undesired non-perovskite polymorph at room temperature [37]. In order to study the stability of CsPbX_3_@Cu-BDC under continuous irradiation, the PL spectrum was measured every two hours. In these three samples, the intensity of photoluminescence spectrum was almost unchanged (Figure S10). The better phase stability of CsPbX_3_ (X = Cl, Br, and I) quantum dots due to the quantum dot-induced size effects.

## 4. Conclusions

In conclusion, we demonstrated a facile and simple route to fabricate the sub-2 nm CsPbX_3_ perovskite quantum dots by a MOF-confined strategy. The highly uniform microporous structure of MOF can effectively restrict the CsPbX_3_ quantum dots aggregation in synthetic process and endow the obtained sub-2 nm CsPbX_3_ quantum dots with well-disperse and excellent stability in ambient air. The PL emission spectra and lifetimes were not decayed after 60 days. It is noteworthy that the size distribution of these CsPbX_3_ quantum dots is well remained in ambient air without any post-treatment. Both the absorption and PL emission peak are blue shifted to shorter wavelengths from that of the bulk materials, due to the quantum confinement effect of CsPbX_3_ quantum dots. Thereby, this effective strategy provides a new opportunity for preparation of ultra-small photoluminescence quantum dots through confinement effect of MOF.

## Supplementary Materials

The following are available online at https://www.mdpi.com/2079-4991/9/8/1147/s1, Further SEM/FT-IR/XRD/BET characterization of the as-prepared Cu-BDC. EDS spectra and XPS of CsPbCl_3_@Cu-BDC, CsPbBr_3_@Cu-BDC and CsPbI_3_@Cu-BDC. Figure S1 (a) The SEM of Cu-BDC. (b) FT-IR spectra of Cu-BDC, Figure S2 The XRD of Cu-BDC and simulated Cu-BDC, Figure S3 (a) N2 adsorption isotherms and (b) pore size distribution analyses for Cu-BDC, Figure S4 Theoretical structure diagram of Cu-BDC, Figure S5 The EDS spectra of CsPbCl3@Cu-BDC, Figure S6 The EDS spectra of CsPbBr3@Cu-BDC, Figure S7 The EDS spectra of CsPbI3@Cu-BDC, Figure S8 The XPS total spectra about CsPbCl_3_@Cu-BDC, CsPbBr_3_@Cu-BDC, CsPbI_3_@Cu-BDC, Figure S9 (a) Respectively XPS spectra of the CsPbCl_3_@Cu-BDC quantum dots for Cs 3d, Pb 4f, Cl 2p. (b) Respectively XPS spectra of the CsPbBr_3_@Cu-BDC quantum dots for Cs 3d, Pb 4f, Br 3d. (c) Respectively XPS spectra of the CsPbI_3_@Cu-BDC quantum dots for Cs 3d, Pb 4f, I 3d, Figure S10 The stability of PL emission response of CsPbCl_3_@Cu-BDC, CsPbBr_3_@Cu-BDC and CsPbI_3_@Cu-BDC over 8 h. Table S1 The elemental ratio of CsPbCl3@Cu-BDC, CsPbBr3@Cu-BDC, CsPbI3@Cu-BDC testing by EDS point scanning, Table S2 The XPS elemental ratio of CsPbCl_3_@Cu-BDC, CsPbBr_3_@Cu-BDC, CsPbI_3_@Cu-BDC, Table S3 The atomic molar ratio of the of CsPbCl_3_@Cu-BDC, CsPbBr_3_@Cu-BDC, CsPbI_3_@Cu-BDC, Table S4 Fitting parameters of the PL decay curve of CsPbCl_3_@Cu-BDC, CsPbBr_3_@Cu-BDC, CsPbI_3_@Cu-BDC NCs.
