# Supplementary material for: MOF-Confined Sub-2 nm Stable CsPbX3 Perovskite Quantum Dots
Source: Nanomaterials (Basel). 2019 Aug 10;9(8):1147. doi: 10.3390/nano9081147 (PMC6723177; doi:10.3390/nano9081147)
Supplement: Supplementary file 1 [file nanomaterials-09-01147-s001.pdf]

MOF-Confined Sub-2 nm Stable $CsPbX_3$ Perovskite Quantum Dots

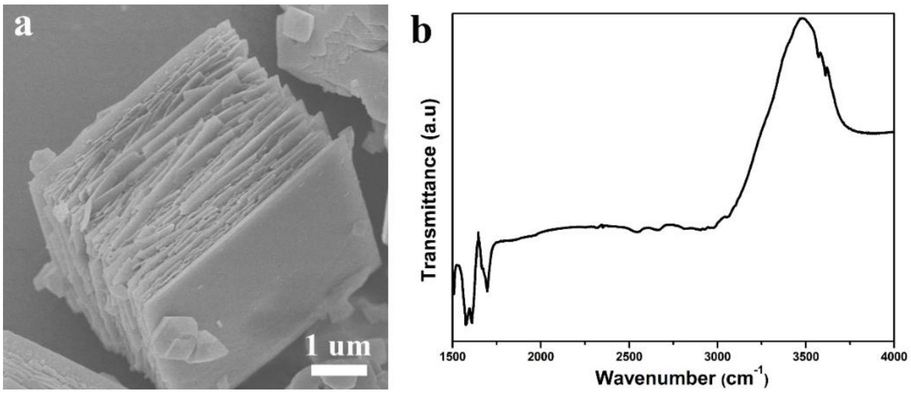

Figure S1 (a) The SEM of Cu-BDC. (b) FT-IR spectra of Cu-BDC.

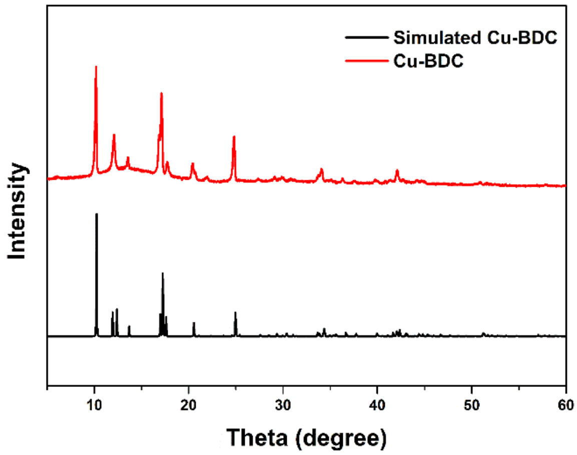

Figure S2 The XRD of Cu-BDC and simulated Cu-BDC.

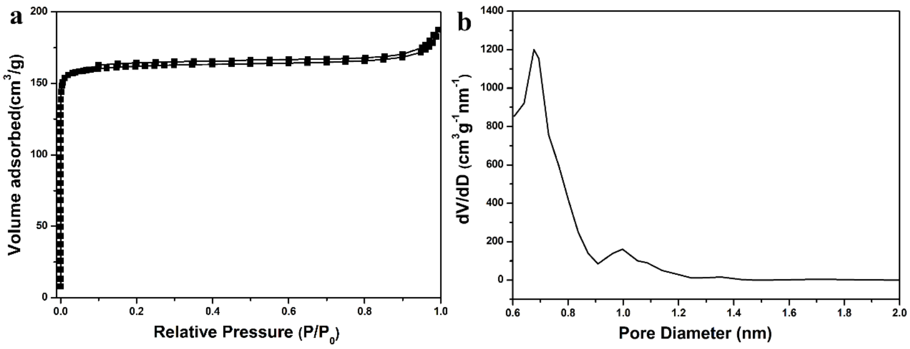

Figure S3 (a) N_2 adsorption isotherms and (b) pore size distribution analyses for Cu-BDC.

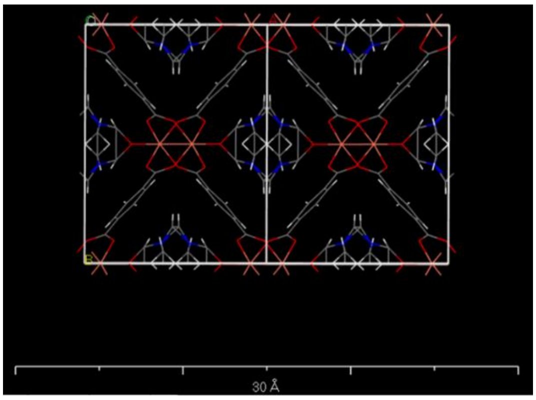

Figure S4 Theoretical structure diagram of Cu-BDC.

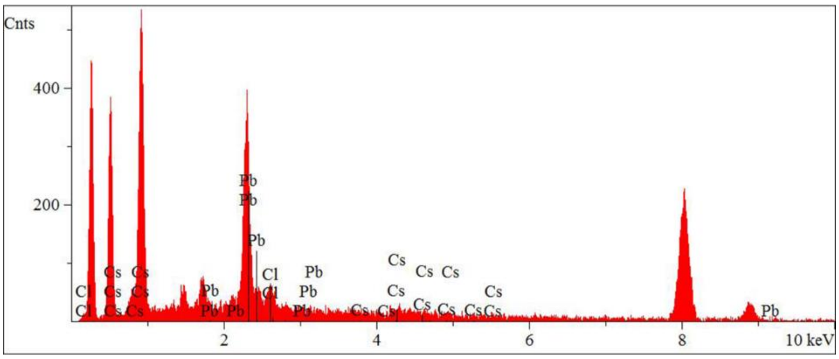

Figure S5 The EDS spectra of $CsPbCl_3$@Cu-BDC.

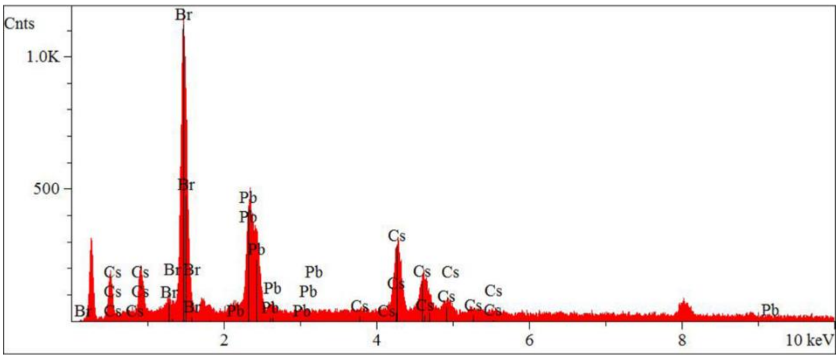

Figure S6 The EDS spectra of $CsPbBr_3$@Cu-BDC.

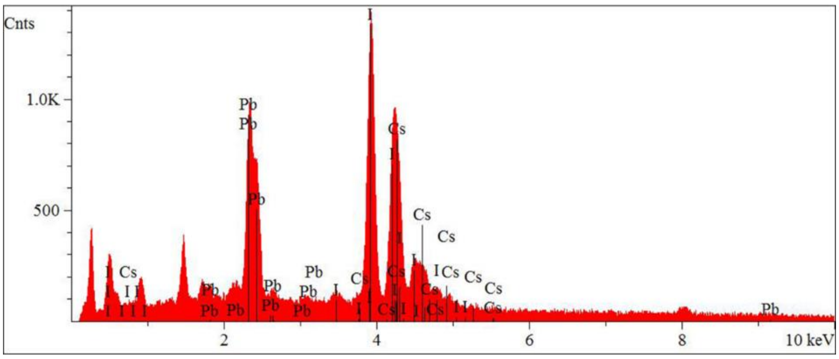

Figure S7 The EDS spectra of $CsPbI_3$@Cu-BDC.

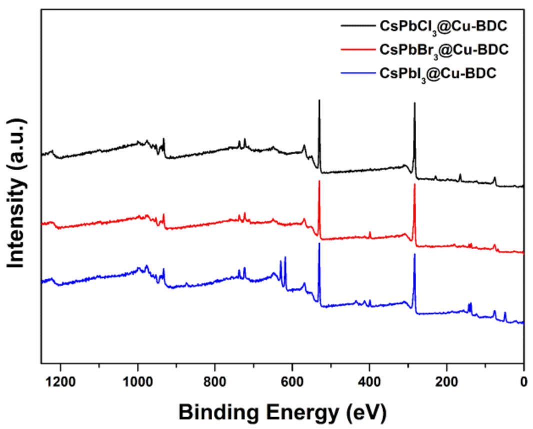

Figure S8 The XPS total spectra about $CsPbCl_3$@Cu-BDC, $CsPbBr_3$@Cu-BDC, $CsPbI_3$@Cu-BDC.

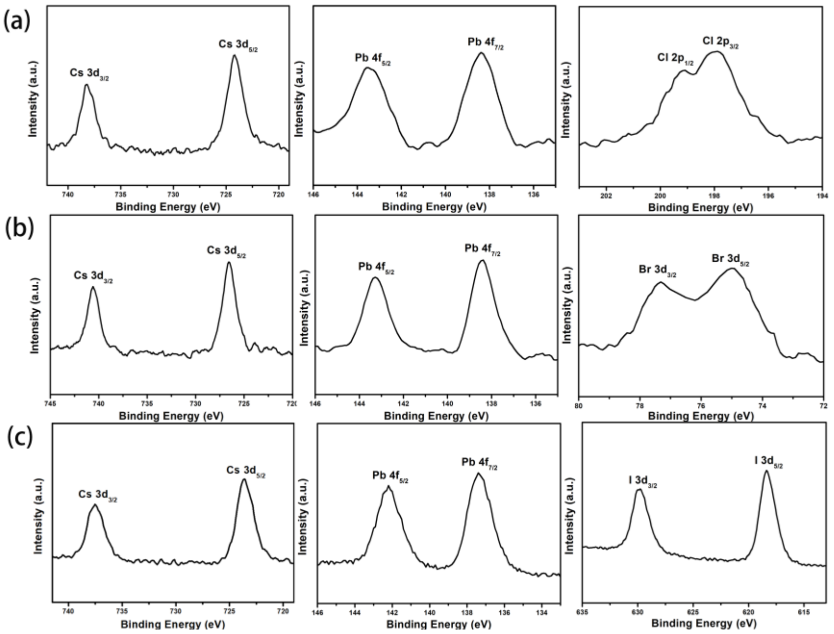

Figure S9 (a) Respectively XPS spectra of the $CsPbCl_3$@Cu-BDC quantum dots for Cs 3d, Pb 4f, Cl 2p. (b) Respectively XPS spectra of the $CsPbBr_3$@Cu-BDC quantum dots for Cs 3d, Pb 4f, Br 3d. (c) Respectively XPS spectra of the $CsPbI_3$@Cu-BDC quantum dots for Cs 3d, Pb 4f, I 3d.

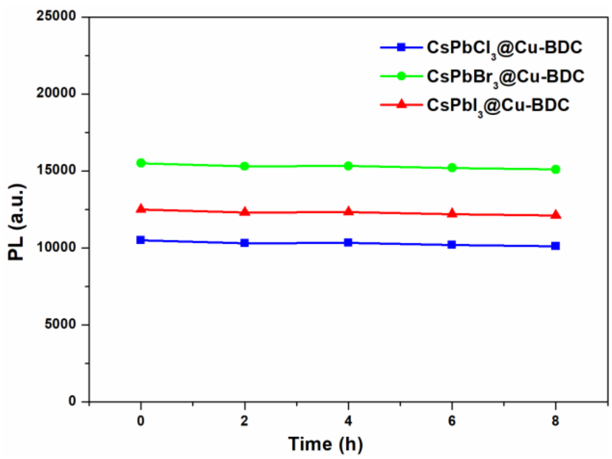

Figure S10 The stability of PL emission response of $CsPbCl_3$@Cu-BDC, $CsPbBr_3$@Cu-BDC and $CsPbI_3$@Cu-BDC over 8 h.

Table S1 The elemental ratio of $CsPbCl_3$@Cu-BDC, $CsPbBr_3$@Cu-BDC, $CsPbI_3$@Cu-BDC testing by EDS point scanning.

Samples	Cs	Pb	Cl/Br/I
$CsPbCl_3$@Cu-BDC	23.51%	22.09%	54.40%
$CsPbBr_3$@Cu-BDC	23.40%	19.10%	57.49%
$CsPbI_3$@Cu-BDC	18.29%	19.56%	62.15%

Table S2 The XPS elemental ratio of $CsPbCl_3$@Cu-BDC, $CsPbBr_3$@Cu-BDC, $CsPbI_3$@Cu-BDC.

Samples	Cs	Pb	Cl/Br/I
$CsPbCl_3$@Cu-BDC	23.56%	21.12%	55.32%
$CsPbBr_3$@Cu-BDC	21.95%	19.96%	58.09%
$CsPbI_3$@Cu-BDC	24.01%	18.21%	57.78%

Table S3 The atomic molar ratio of the of $CsPbCl_3$@Cu-BDC, $CsPbBr_3$@Cu-BDC, $CsPbI_3$@Cu-BDC.

Samples	Cs	Pb
$CsPbCl_3$@Cu-BDC	1	0.95
$CsPbBr_3$@Cu-BDC	1	1
$CsPbI_3$@Cu-BDC	1	1.06

The quantum yield of PLQY was calculated by measuring the integrated PL intensity and Cu-BDC as a standard one.

The decay curves were fitted using a tri-exponential decay kinetic:

$$\mathrm{A(t)} = A_1 e^{(-\frac{t}{\tau_1})} + A_2 e^{(-\frac{t}{\tau_2})} + A_3 e^{(-\frac{t}{\tau_3})}$$

and the average PL lifetimes (τaverage) are calculated by the following equation:

$$\tau_{average} = (A_1\tau_1^2 + A_2\tau_2^2 + A_3\tau_3^2)/(A_1\tau_1 + A_2\tau_2 + A_3\tau_3)$$

Table S4 Fitting parameters of the PL decay curve of $CsPbCl_3$@Cu-BDC, $CsPbBr_3$@Cu-BDC, $CsPbI_3$@Cu-BDC NCs.

Sample	A1(%)	A2(%)	A3(%)	τ1(ns)	τ2(ns)	τ3(ns)	τavg(ns)	PLQY(%)
CsPbCl3@Cu-BDC	71.05	21.45	7.5	0.64	3.08	23.39	15.1±0.2	4.12±0.5
CsPbBr3@Cu-BDC	5.39	33.88	61.74	1.73	7	26.93	24.4±1.3	9.96±0.3
CsPbI3@Cu-BDC	20.73	27.51	51.77	0.48	2.69	20.04	18.75±0.6	18.30±0.2
